# Life History Traits and Longevity of the Invasive Asian Common Toad *Duttaphrynus melanostictus* (Schneider, 1799) in Madagascar

**DOI:** 10.3390/ani13132099

**Published:** 2023-06-24

**Authors:** Fabio Maria Guarino, Franco Andreone, Marcello Mezzasalma, Fulvio Licata, Simona Puoti, Bárbara Santos, Walter Cocca, Jean Francois Solofoniaina Fidy, Serge Herilala Ndriantsoa, Jean Noel, Tsanta Fiderana Rakotonanahary, Rodino Fetrarijahona Harison, Gaetano Odierna, Angelica Crottini

**Affiliations:** 1 Department of Biology, University of Naples Federico II, Via Cinthia 26, I-80126 Naples, Italy; fabio.guarino@unina.it (F.M.G.); si.puoti@studenti.unina.it (S.P.); gaetanodierna@gmail.com (G.O.); 2 Museo Regionale di Scienze Naturali, Via G. Giolitti, 36, I-10123 Torino, Italy; 3 Department of Biology, Ecology and Earth Science (DiBEST)—University of Calabria, Via P. Bucci 4/B, I-87036 Rende, Italy; 4 CIBIO, Centro de Investigação em Biodiversidade e Recursos Genéticos, InBIO Laboratório Associado, Campus de Vairão, Universidade do Porto, 4485-661 Vairão, Portugal; barbarasantosbio@gmail.com (B.S.); walter.cocca85@gmail.com (W.C.); tiliquait@yahoo.it (A.C.); 5 BIOPOLIS Program in Genomics, Biodiversity and Land Planning, CIBIO, Campus de Vairão, 4485-661 Vairão, Portugal; 6 Departamento de Biologia, Faculdade de Ciências, Universidade do Porto, Rua do Campo Alegre s/n, 4169-007 Porto, Portugal; 7 Madagascar Fauna and Flora Group, BP 442, Toamasina 501, Madagascar; rendrirendry@gmail.com (J.F.S.F.); karenlmfreeman@gmail.com (J.N.); 8 Département de Biologie Animale, Université d’Antananarivo, BP 906, Antananarivo 101, Madagascar; nsehel2006@gmail.com; 9 Turtle Survival Alliance, BP 8511, Antananarivo 101, Madagascar; tsantafiderana@gmail.com; 10 Institut Supérieur de Science, Environnement et Développement Durable (ISSEDD), Université de Toamasina, Toamasina 501, Madagascar; rijashalom501@gmail.com

**Keywords:** Amphibia, invasive species, skeletochronology, age estimation, gonadal analysis, age at sexual maturity

## Abstract

**Simple Summary:**

The Asian common toad *Duttaphrynus melanostictus* represents a serious threat to the biodiversity of Madagascar, where it was introduced in around 2010. Here, we study some traits of its life history, including its body size, age structure, and age at sexual maturity based on individuals sampled at different sites of Toamasina, on the east coast of Madagascar, in 2016, 2018, and 2019. The individuals studied here were larger, although no longer-living, with respect to individuals of the native populations in SE Asia. In the invasive population of Madagascar, the males were significantly smaller and younger than the females when data from different sites were pooled. However, when data are analyzed separately, this was true only for one site. The maximum age ascertained in this study is 3 years in males and 6 years in females. Males and females reach sexual maturity after the first and second year of age, respectively. Further studies on the life history traits of the population of *D. melanostictus* in Madagascar can be relevant for the management of this invasion as well as to better understand the biology of the species.

**Abstract:**

We analyzed the body length, age structure, and age at sexual maturity of the invasive Asian common toad *Duttaphrynus melanostictus* from different sites in Toamasina, east Madagascar. We used skeletochronology as a proxy for age estimation, while gonads were histologically analyzed to determine the age of sexual maturity. The analysis of pooled age data from three sites investigated in 2016 showed that both sexes were larger, although not older, than those of native populations. For the individuals from Madagascar, the males were significantly smaller and younger (mean ± SD, SVL: 71.4 ± 1.6 mm; age: 1.8 ± 0.7 years) than the females (SVL: 78.42 ± 1.9 mm; age: 2.7 ± 1.3 years), when the data were pooled, but when the data were analyzed separately for each of the three sites, similar results were obtained only for one site. The oldest recorded male and female were 3 and 6 years old, respectively. Gonadal histology showed that the males and females reach sexual maturity after the first and second years of age, respectively. Further studies are needed to understand if the larger size and faster growth rates observed in the invasive population of *D. melanostictus* in Madagascar are a consequence of more favorable environmental conditions with respect to the native range (e.g., the availability of larger trophic niches, a lack of competitors, and lower predatory pressure), and we suggest to extend the monitoring of these life history traits to understand how they might influence the invasion.

## 1. Introduction

Amphibians and reptiles are establishing as model organisms due to their high species richness and diversity and have been widely studied in different fields such as genetics, biogeography, and more recently invasion biology [1,2,3,4]. Due to their species richness and diversity, several areas of the world remain poorly characterized and more efforts should be invested in these places.. One of these areas is indeed Madagascar, which is characterized by an incredibly rich amphibian fauna [5].

Unluckily, Madagascar is currently the stage of an ongoing invasion determined by an introduced amphibian species: the Asian common toad *Duttaphrynus melanostictus* (Schneider, 1799) (Amphibia: Anura: Bufonidae). This species is native to Southeast Asia and was introduced to Madagascar between 2007 and 2010 [6,7,8,9,10,11]. This species is considered the third most harmful alien amphibian species, after *Rhinella marina* and *Xenopus laevis* [1], and represents a serious threat to the unique biodiversity of Madagascar, as well as to the biodiversity of other geographical areas where it was introduced [12,13]. Indeed, several biological (including the presence of parotid glands which produce bufotoxins) and ecological (including high clutch size and a generalist diet) characteristics allow this species to readily adapt to new environments, be competitive with native species, have disruptive effects on predators, and establish stable populations at faster rates [11,14]. Recent studies have revealed a drastic impact on the native Malagasy frog-eating snake *Madagascarophis colubrinus*, with an estimated monthly mortality rate due to toad poisoning of ca. 5%, which could lead to local extinction of this species if the impacts remain constant over time [14]. According to Vences et al. [15], the invasive population of *D. melanostictus* in Madagascar originated from SE Asia, namely Vietnam and/or Cambodia, likely from a single source of introduction. In this study, we investigated life history traits, including body length, age structure, and age at sexual maturity, of this invasive population using skeletochronology and histological analysis of the gonads. This information is crucial to informing management strategies and minimizing the impacts of this invasion.

## 2. Materials and Methods

### 2.1. Sampling

**Figure 1:**
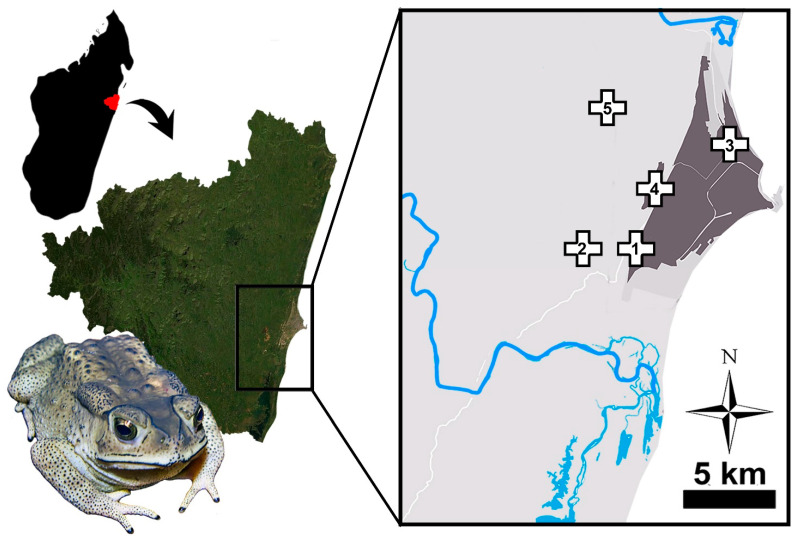
Geographic position of Toamasina (**left**) and the five sampling sites (**right**).

A total of 101 toads were sampled at 5 sites around the city of Toamasina (east coast of Madagascar), hereby called: site 1 (18.20195° S, 49.33701° E), site 2 (18. 21342° S, 49.30854° E), site 3 (18.12476° S, 49.39223° E), site 4 (18.14651° S, 49.36406° E), and site 5 (18.13951° S, 49.34605° E) (Figure 1). The toads were searched for at night and collected by hand in September 2016 (sites 1–3), and between October 2018 and May 2019 (sites 4–5). Thirty-five individuals at site 1, 26 at site 2, 20 at site 3, 9 at site 4, and 11 at site 5 were collected (see Tables S1 and S2). A detailed description of sampling sites 1–3 can be found in Santos et al. [16], while that of the sites 4 and 5 can be found in Licata et al. [17]. The climate of the study area is characterized by monthly stable temperatures (ranging from 21 °C in July and 27 °C in February) and by the occurrence of alternating dry (between May and September) and moist (between October and April) seasons [18].

After capture, the stage of development (juvenile or adult), sex (based on the secondary sexual characteristics; see [19,20]), and snout–vent length (SVL) were collected (Tables S1 and S2). SVL measurements were collected using a caliper to the nearest 0.1 mm. The animals were anesthetized and euthanatized with 250 mg/L tricaine methane–sulfonate (MS-222, SIGMA).

### 2.2. Skeletochronology

For age estimation, we applied standard skeletochronological procedures [21,22,23] on the phalanges of 55 toads from sites 1–3 (12 males, 13 females, and 4 juveniles from site 1; 8 males and 10 females, from site 2; 2 males, 5 females, and 1 juvenile from site 3) (Table S1). From a sub-sample of randomly selected toads from sites 1–3 (*n* = 26), we also processed sections of the femur to verify the precision of the skeletochronology methods applied to the phalanges (Table S1). The phalanges and femurs were cleaned from soft tissues and preserved in 75% ethanol. They were decalcified in 5% nitric acid for 1.5–2 h (phalanges) or 3–4 h (femurs), washed under running tap water for 12 h, dehydrated through a series of graded ethanol baths, cleared with a terpene of natural origin (Bioclear, Bio Optica, Milan, Italy), and embedded with paraffin (melting point 60 °C). Diaphyseal bone cross-sections (12 μm-thick for the phalanges and 15 μm-thick for the femurs) were cut using a standard rotative microtome. The sections were stained with Mayer’s hematoxylin for 30 min and then dehydrated in the open air and mounted in synthetic medium (Bio Mount HM, Bio Optica, Milan). The stained sections were examined using a Motic BA340 light microscope, equipped with a digital camera. The count of the lines of arrested growth (LAGs) was performed independently by two observers (FMG and MM) on at least ten sections per individual and without prior knowledge of the SVL and sex of the analyzed individual. According to other studies [22,24,25], we selected diaphyseal sections with the smallest medullar cavity and the widest periosteal bone, so that the total resorption of the innermost (oldest) periosteal LAG was less likely to have occurred. The number of completely resorbed LAGs was assessed based on the comparison between the phalangeal sections at the same magnification from different individuals. Age was estimated by counting the visible LAGs with the addition of any resorbed ones.

We assumed that a LAG is formed each year during the cooler and drier period (July–September), when the temperature and rainfall are lower and food availability is expected to be less abundant.

The growth rate was estimated using von Bertalanffy’s model, as previously performed in several other anuran studies [22,26,27,28]. The general form of von Bertalanffy’s growth equation used was: SVL*t* = SVL ∞ (1 − *e^k^*^(t–t0)^), where SVL*t* is the body length at age *t*; SVL∞ is the estimated maximum body length; *e is* the base of the natural logarithm; *k* is the growth coefficient; and *t*0 is the age at metamorphosis, which in amphibians represents the starting point of the growth interval. We considered 9.4 mm as the mean size at metamorphosis according to Mogali et al. [29]. Estimates of SVLmax and K with the corresponding 95% confidence interval were computed using Growth II software [30].

### 2.3. Sexual Maturity

To assess the age at sexual maturity, we analyzed the gonads of five specimens from sites 1–3 which were also processed for skeletochronology (two males, SVL 55.3 and 63.2 mm; three females, SVL 81.2, 95.5, and 100.9 mm) (see Table S1). Furthermore, the gonads of 20 specimens collected from sites 4–5 (six males, SVL range: 43.70–56.0 mm; fourteen females, range 58.1–79.5 mm) (see Table S2) were also analyzed to confirm the minimum body size for the onset of sexual maturity according to [31]. The gonads were fixed in neutral buffered formalin and embedded in paraffin following standard protocols [23]. They were sectioned at 7 μm thickness using a rotary microtome. The sections were stained with Mallory Trichrome (Bioptica, Milano), dehydrated through a series of graded ethanol baths, cleared with a terpene of natural origin (Bioclear, Bio Optica, Milan, Italy), and mounted in synthetic medium (Bio Mount HM, Bio Optica, Milan). The stained sections were observed under a Motic BA340 light microscope equipped with a digital camera. In the males, the presence of different germ cell stages in the seminiferous tubules was assessed according to [32], and only individuals in advanced spermatogenesis, including spermatids and sperm, were considered mature. Female sexual maturity and reproductive activity were confirmed by the presence of yolked follicles and oviductal eggs [32,33].

### 2.4. Statistical Analysis

The descriptive statistics included the mean ± standard deviation, minimum and maximum. For the age modal, values were also given. Since several subsets of SVL and age data differed from a normal distribution or included small sample sizes, we used the non-parametric Kruskal–Wallis test and Mann-Whitney U test to test for significant differences between the two sexes and sites. The relationship between size and age was assessed with Spearman correlation. The significance level was set at 0.05. Statistical analysis was performed using PAST software version 3.22 [34].

## 3. Results

### 3.1. Body Length

The measurements of the SVL of *D. melanostictus* individuals from sites 1–3 and the pooled data set are given in Table 1. The mean SVL of the males, as well as that of the females, did not significantly differ between the three sites (Kruskal–Wallis test, males: H = 2.78, *p* = 0.35; females: H = 4.4, *p* = 0.11). The mean SVL of the males was significantly lower than that of the females for the pooled data set (Mann–Whitney, Z = 2.11, *p* = 0.03). At site 3, the juveniles were the dominant cohort and included four very small-sized juveniles (25–35 mm SVL).

### 3.2. Skeletochronological Observations

**Figure 2:**
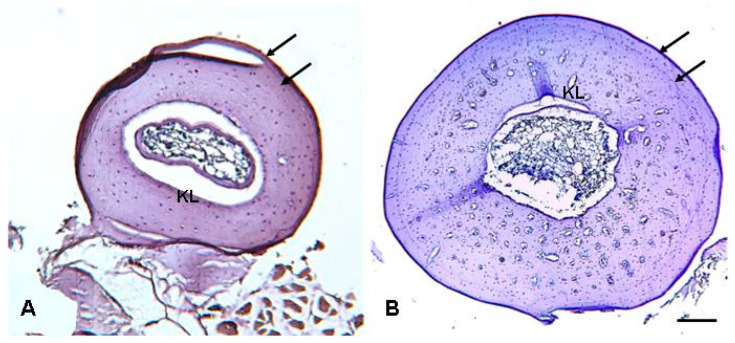
Representative cross-sections of the phalanx (**A**) and femur (**B**) taken from a *D. melanostictus* female, laboratory label ACZC12131, SVL: 77.9 mm. Arrows indicate the lines of arrested growth (LAGs), of which the outermost is confluent with the outer bone cortex margin. KL: Katschenko line. Scale bar: 130 µm in (**A**) and 320 µm in (**B**).

**Figure 3:**
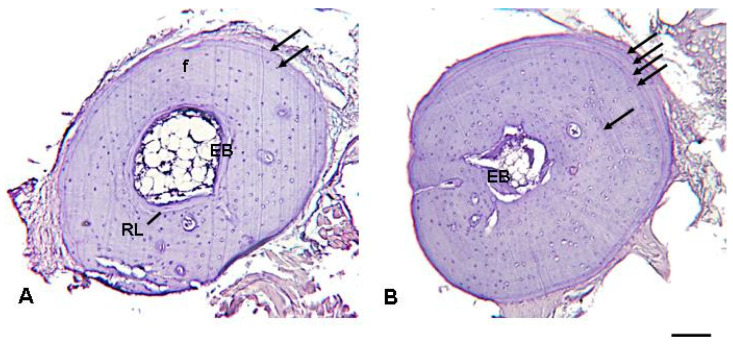
Representative cross-sections of the phalanx of a *D. melanostictus* (**A**) male, laboratory label ACZC12157, SVL: 70 mm; (**B**) female, laboratory label ACZC12173, SVL: 95.5 mm. Arrows indicate lines of arrested growth (LAGs). In (**B**), the faint multiple innermost circular haematoxynophilic lines were interpreted as true LAGs. EB: endosteal bone; f: false line; RL: reversal line. Scale bar: 150 µm in (**A**) and 180 µm in (**B**).

Hematoxynophilic periosteal lines were always observed in the phalanx and femur diaphyseal sections and showed full correspondence in the specimens where it was possible to analyze both types of bone, indicating that skeletochronological reading can be successfully performed on either the phalanx or femur (Figure 2). The hematoxynophilic line at the edge of the medullary cavity was interpreted as a Katschenko line (KL) according to other studies [35,36]; the remaining circular periosteal lines are typical lines of arrested growth (LAGs), although their distinctiveness was very variable among the different sections and individuals (Figure 3). In the phalangeal sections of five individuals (about 9% of the whole sample), the LAGs were faint and less clear, sometimes confused with the local lamellar organization of the periosteal cortex.

Endosteal remodeling phenomena were observed, particularly in larger (>80 mm) and likely older animals, as evidenced by the presence of conspicuous endosteal bone that had eroded the periosteal cortical layer. By comparing the diameter of the medullary cavity of the smallest individuals in the study sample (<50 mm) with those of the largest individuals (whose sections showed signs of endosteal remodeling after qualitative histological examination), it emerged that the first (innermost) LAG was sometimes (15% of the study sample) partially removed by bone remodeling.

### 3.3. Age

Of the 55 specimens, 5 individuals showed unclear LAGs and were excluded from the analyses.

The mean and modal age (in years) of *D. melanostictus* from Sites 1–3 and for the pooled data set is given in Table 2. The mean age of the males, as well as that of the females, did not significantly differ between the three sites (Kruskal–Wallis test, males: H = 3.41, *p* = 0.13; females: H = 0.91, *p* = 0.61). The mean age of the males was significantly lower than that of the females for the pooled data set (Mann–Whitney, Z = 2.36, *p* = 0.01) and significantly lower at site 1 (Mann-Whitney, Z = 1.90, *p* < 0.05) but not for site 2 (Mann–Whitney, Z = 1.18, *p* = 0.23). At site 3, the sampling size was too low to apply statistics. For the pooled data set, the males and females exhibited unimodal (2 years) and bimodal age (2 and 3 years), respectively.

**Figure 4:**
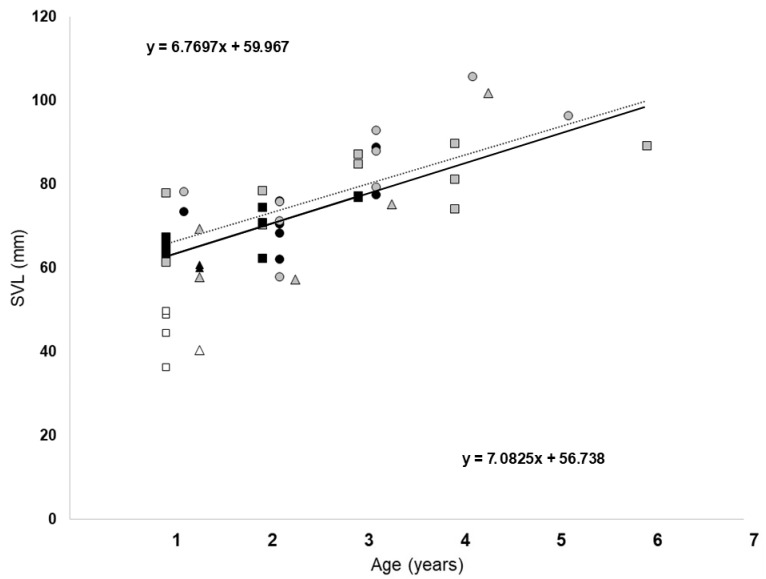
Relationships between age (years) and SVL in *D. melanostictus* individuals from Madagascar. Squares, circles, and triangles represent toads from sites 1, 2, and 3, respectively. The black, gray, and empty symbols refer to males, females, and juveniles, respectively. The solid line and the dotted line represent the linear regression line for males and females, respectively, for the pooled data. A linear regression equation is also given for the males (bottom) and females (top).

The relationship between age and SVL is shown in Figure 4. Age was positively correlated with SVL in both sexes when we analyzed the pooled data set (Spearman’s rank correlation, males rs  =  0.74, *p*  <  0.001; females rs  =  0.73, *p*  <  0.001), but only for the females of site 1 (Spearman’s rank correlation, rs  =  0.65, *p*  = 0.02) and 2 (Spearman’s rank correlation, rs  =  0.84, *p*  = 0.006) when the data were analyzed separately. The ANCOVAs showed that the difference in the linear regression line between the sexes was not significant, (F_1,43_ = 0.95. *p* = 0.332) using age as a covariate. Von Bertalanffy’s growth model using pooled data showed that for both sexes, the estimated asymptotic SVL was smaller than the maximum SVL recorded (SVL∞ ± CI, males: 75.48 ± 0.42; females: 87.52 ± 0.43). The growth coefficient significantly differed between sexes (k ± CI, males: 1.04 ± 0.02; females: 1.59 ± 0.05).

### 3.4. Assessment of Gonad Status and Individual Age

**Figure 5:**
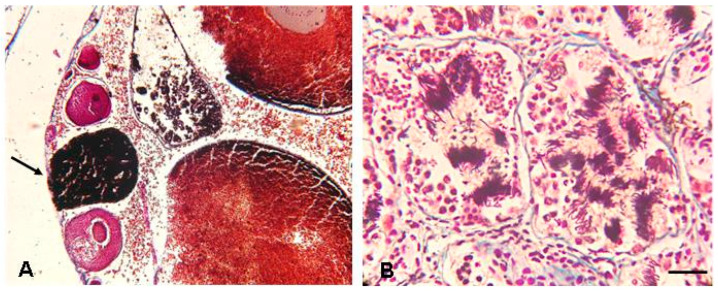
Histological sections of gonads of *D. melanostictus* from Madagascar stained with Mallory’s trichrome. (**A**) Ovary of a female (SVL: 84.6 mm) with late vitellogenic follicles and atresia (arrow); (**B**) testis of a male (SVL: 55.2 mm) with all spermatogenetic stages. The bar corresponds to 700 µm in (**A**) and 200 µm in (**B**).

The two females (81.2 and 95.5 mm), analyzed for both skeletochronology and gonad histology (see Table S1), had an estimated age of 4 and 5 years, respectively, and their ovaries contained many postvitellogenetic and late vitellogenetic follicles. One female measuring 100.9 mm also had postvitellogenetic and late vitellogenic follicles, but it was not possible to estimate its age. The ovaries of all adult females were characterized by the presence of numerous atretic follicles (Figure 5A). The two males (55 and 63.2 mm), analyzed for both skeletochronology and gonad histology, had an estimated age of 1 year and testicles with all spermatogenetic stages, including sperm (Figure 5B). Among the specimens from sites 4–5 (see Table S2), the females ranging in SVL from 56.9 to 66.8 mm (*n* = 4) had previtellogenetic follicles, and all females with SVL ranging from 69.9 to 79.5 mm (*n* = 11) had follicles in vitellogenetic growth, thus indicating that they were sexually mature. The males ranging between 43.4 and 48.4 mm (*n* = 5) showed testicles with only spermatogonia, while one male with SVL of 56 mm had testicles with all spermatogenetic stages, including sperm (see Table S2).

## 4. Discussion

Species’ life history traits can be mismatched between invasive and native populations due to different environmental conditions (e.g., a lack of competitors and lower predatory pressure). On the other hand, trait mismatches can result from pre-existing differences in the native source populations and can emerge during acclimatization through genetic drift and local adaptation [37,38]. The study of life history traits, including growth rate, age, and size at maturity, of an invasive population can be useful for understanding how each trait may have contributed to successfully colonizing a new area.

In accordance with previous studies [11], males of the invasive population of the Asian common toads in Madagascar are larger in body size than the native populations, However, we did not find the same conclusion as far as the females are concerned, which were found to be slightly smaller. Indeed, the mean SVL ± SD of the adults and subadults was 71.9 ± 1.6 mm in males and 78.2 ± 1.9 mm in females in Madagascar and 60.7 ± 6.2 mm in males and 81.3 ± 9.2 in females in a native population from Vietnam [31]. Unlike what is known for native populations of Asian toad [31] as well as for other bufonids (e.g., *Bufo bufo* [39], *Sclerophrys pardalis* [40], and *Rhinella arenarum* [28]), the females were not always significantly larger than the males. This finding is in agreement with Licata et al. [11], where it has been suggested that the sexual dimorphism in the size of invasive Asian toads in Madagascar is reduced compared to that of native populations. Our data suggest that the larger size of the invasive common Asian toad of Madagascar is not dependent on greater longevity, as shown by our skeletochronological data (see below), and this might be related to local differences in food availability [11]. The prevalence of juveniles at Site 3, which at the time of sampling in 2016 was at the invasion front, suggests that the dispersal front is mainly formed by young toads [10,41].

Skeletochronology is one of the most used and reliable indirect methods for age determination in amphibians and reptiles [42,43,44,45]. In accordance with previous studies [46,47,48,49], our study shows the suitability of this method for the invasive population of this species as well, even though some difficulties were encountered in the interpretation of the results. The first (innermost) hematoxynophilic mark was often less stained than those successively layered, but it was considered here as a true LAG, based on its position with respect to the medullary cavity. The distinctiveness of growth marks is known to reflect the amplitude of the growth cycles and the severity of inactivity both at individual and population levels [50,51]. The visibility of the LAGs was a major problem, with considerable differences in the LAGs’ distinctiveness both in different sections of the same individual and between the individuals. As previously proposed [27], this seems to be an individual, age-dependent phenomenon, likely related to microclimatic conditions and the length and intensity of the torpor period. This period in eastern Madagascar probably coincides with the dry season (from May to September), when more unfavorable environmental conditions (e.g., less available food) can cause some continuous period of inactivity of the individuals.

So far, a skeletochronological estimate of the age of *D. melanostictus* has been provided for populations inhabiting different areas of India [46,47,48,49]. These studies suggest that the species generally live for a maximum of four [47], five [48], or six [49] years and only reaches nine years in exceptional cases [48]. Based on the analysis of the published phalangeal sections [46], we consider the record age of 12 years (estimated for a female) dubious because we think that some of the LAGs are probably lamellae of the endosteal bone (see Figure 2B) [46]. In our study, the estimated maximum age was three years in males and six years in females, which is in general agreement with previous skeletochronological studies [47,49]. Overall, these data indicate that *D. melanostictus* is not a very long-lived species, especially if compared with other bufonids from temperate regions, such as *Bufo bufo* [39] and *Bufotes variabilis* (=*B. viridis*) [52]; although, our results are in agreement with previous studies that found that tropical or sub-tropical species are shorter-lived and mature earlier than species from temperate areas [53,54,55]. However, we cannot exclude that there may not have been enough time for the introduced population to achieve longer life spans due to the recent introduction of this species to Madagascar [8,9,56], and further studies are needed to confirm the maximum age of the individuals of this invasive population.

In agreement with Kumbar et al. [48], we observed a positive correlation between body length and age in both sexes, although this result was significant only with pooled data. When the data were analyzed separately for each site, the positive correlation was significant only for females from sites 1 and 2. This is probably due to the small sample size, rather than a true variability in both time and space [57]. However, in each site and for both sexes, we found very different growth rates, as confirmed by the wide overlap of SVLs among the different age classes. Furthermore, the fact that the male toads seem to be larger than their native conspecifics despite having comparable maximum age suggests that the former have higher growth rates.

By combining skeletochronological age estimation with gonad analysis, the present study shows that males and females of the *D. melanostictus* population introduced to Madagascar attain sexual maturity when they show one and two LAGs, respectively. This result suggests that sexual maturity is achieved when males are one years old and when females are two years old, in agreement with published data [49].

## 5. Conclusions

This study analyzes data on the body length, age structure and age at sexual maturity of the invasive Asian common toad *D. melanostictus* from different sites in Toamasina (east Madagascar). It provides useful information to better understand the life history of this species, in particular when considered as a harmful species in a biodiversity-rich context such as Madagascar, and it is also considered as a relevant threat by the “A Conservation Strategy for the Amphibians of Madagascar” project and consequent Sahonagasy Action Plans [58,59], that have been proposed over the last two decades.

Our findings show that the *D. melanostictus* males introduced in Madagascar are on average larger than the native males, although they, as well as the introduced females, do not seem to live longer (males’ maximum age: 3 years; females’ maximum age: 6 years). Similar to the native populations, males and females attain sexual maturity in their first and second year after metamorphosis and exhibit a female-biased size dimorphism, although reduced when compared to native populations. Overall, the life history traits examined here largely overlap with those of native populations. Further studies are needed to understand if the larger size and faster growth rates observed in the invasive population in Madagascar are a consequence of more favorable environmental conditions, with respect to the native range (e.g., larger trophic niches, a lack of competitors, and lower predatory pressure). This aspect can have several consequences on the management of the invasive population, and we encourage the development of further studies on this matter.

## Figures and Tables

**Table 1:** SVL (in mm) of *D. melanostictus* at sampling sites 1–3 and for the pooled data set. Mean ± standard deviation (SD) and the min and max values are reported. N: number of sampled individuals; ND: phenotypically unsexed.

	Site 1	Site 2	Site 3	Pooled Sites
Males	73.4 ± 2.1	70.9 ± 2.3	67.5 ± 8.4	71.4 ± 1.6
	62.2–89.8	60.0–88.2	58.8–88.4	59.8–89.8
	*n* = 15	*n* = 11	*n* = 3	*n* = 29
Females	81.5 ± 2.2	78.7 ± 3.8	71.0 ± 5.6	78.2 ± 1.9
	61.4–89.7	57.5–104.8	57.6–109.0	57.5–104.8
	*n* = 14	*n* = 13	*n* = 7	*n* = 34
Juveniles	45.1 ± 2.0	45.7 ± 4.2	38.5 ± 3.0	41.5 ± 1.9
(ND)	36.2–49.7	41.5–49.9	25.4–49.2	25.4–49.0
	*n* = 6	*n* = 2	*n* = 10	*n* = 18

**Table 2:** Age (in years) of the *D. melanostictus* at the sampling sites 1–3 and for the pooled data set. The mean ± standard deviation (SD) and min and max values are reported. For the modal age of the pooled data, the frequency is also given (between brackets). N: number of sampled individuals; ND: phenotypically unsexed.

	Site 1		Site 2		Site 3		Pooled Sites	
	Mean	Mode	Mean	Mode	Mean	Mode	Mean	Mode
Males	1.7 ± 0.8 1–3 *n* = 9	3	2.1 ± 0.6 1–3 *n* = 8	2	1.0 ± 0 1 *n* = 2	-	1.8 ± 0.7 1–3 *n* = 19	2 (36.8%)
Females	2.9 ± 1.4 1–6 *n* = 12	2, 3, 4	2.7 ± 1.2 1–5 *n* = 9	2, 3	2.2 ± 1.3 1–3 *n* = 5	1	2.7 ± 1.3 1–6 *n* = 26	2, 3 (53.8%)
Juveniles ND	1.0 ± 0 1 *n* = 4	1	- - *n* = 0	-	1 1 *n* = 1	-	1 1 *n* = 5	1

## Data Availability

All the data generated are available within this manuscript and in the supplementary information.

## Supplementary Materials

The following supporting information can be downloaded at: https://www.mdpi.com/article/10.3390/ani13132099/s1, Table S1: Sample of Malagasy *Duttaphrynus melanostiscus* used to estimate snout-vent length (SVL) e for skeletochronology. M = male, F = female; J = Juveniles (undetermined sex). **, individuals whose gonad was also available; Table S2: Specimens of Malagasy *Duttaphrynus melanostiscus* used to evaluate reproductive status. A: adult; F: female; J: juvenile; M: male.
